# Cantilever signature of tip detachment during contact resonance AFM

**DOI:** 10.3762/bjnano.12.96

**Published:** 2021-11-24

**Authors:** Devin Kalafut, Ryan Wagner, Maria Jose Cadena, Anil Bajaj, Arvind Raman

**Affiliations:** 1 School of Mechanical Engineering, Purdue University, West Lafayette, IN 47907, USA

**Keywords:** atomic force microscopy (AFM), contact resonance, nonlinear normal mode (NNM), tip–sample detachment, photothermal excitation

## Abstract

Contact resonance atomic force microscopy, piezoresponse force microscopy, and electrochemical strain microscopy are atomic force microscopy modes in which the cantilever is held in contact with the sample at a constant average force while monitoring the cantilever motion under the influence of a small, superimposed vibrational signal. Though these modes depend on permanent contact, there is a lack of detailed analysis on how the cantilever motion evolves when this essential condition is violated. This is not an uncommon occurrence since higher operating amplitudes tend to yield better signal-to-noise ratio, so users may inadvertently reduce their experimental accuracy by inducing tip–sample detachment in an effort to improve their measurements. We shed light on this issue by deliberately pushing both our experimental equipment and numerical simulations to the point of tip–sample detachment to explore cantilever dynamics during a useful and observable threshold feature in the measured response. Numerical simulations of the analytical model allow for extended insight into cantilever dynamics such as full-length deflection and slope behavior, which can be challenging or unobtainable in a standard equipment configuration. With such tools, we are able to determine the cantilever motion during detachment and connect the qualitative and quantitative behavior to experimental features.

## Introduction

Contact resonance atomic force microscopy (CR-AFM) [1–2], piezoresponse force microscopy (PFM) [3], and electrochemical strain microscopy (ESM) [4] are atomic force microscopy (AFM) [5] methods where the probe tip is held in contact with the sample at a constant average force while a small superimposed vibrational response is monitored. CR-AFM can measure the viscoelastic properties of a sample [6] and observe subsurface features in some biological and electronics samples [7–12]. PFM can measure piezoelectric and ferroelectric properties of a sample [13–16]. ESM can measure the ion diffusion in battery materials [4,17–19]. These different AFM methods provide information on important mechanical and electrical properties across a wide variety of samples.

CR-AFM, PFM, and ESM are built upon the fundamental assumption that as the cantilever is excited, the probe tip remains in contact with the sample. Signal-to-noise ratios in these measurements can be improved by increasing the magnitude of the drive amplitude. However, clear limits on the maximum drive amplitude are insufficiently defined by prior analysis. Moderate drive amplitudes can already result in observance of nonlinearity in the frequency response [20–23], but the most distinct features appear with high drive amplitudes as the fundamental assumption of permanent contact is lost [24–27]. Though nonlinear phenomena in CR-AFM have been connected to the mechanism of detachment in these prior works, less is understood about the dynamic response of the cantilever itself and the connection back to experimental observables in standard AFM configurations.

In this work we present a systematic analytical, numerical, and experimental study of cantilever motion during the process of a probe tip detaching from a sample in CR-AFM. We connect the experimentally observed nonlinear response feature to the onset of tip–sample detachment in our numerical simulations to confirm the conclusions from prior works [26–28]. The simulations allow for deeper insight into cantilever dynamics during the interaction between the AFM probe tip and the sample, which in turn allow us to identify and characterize three different operating regimes: linear, nonlinear softening, and tip–sample detachment. This result provides clear guidance on amplitude limits and why they are observable through cantilever motion monitoring in CR-AFM, PFM, and ESM measurements.

## Results

**Figure 1:**
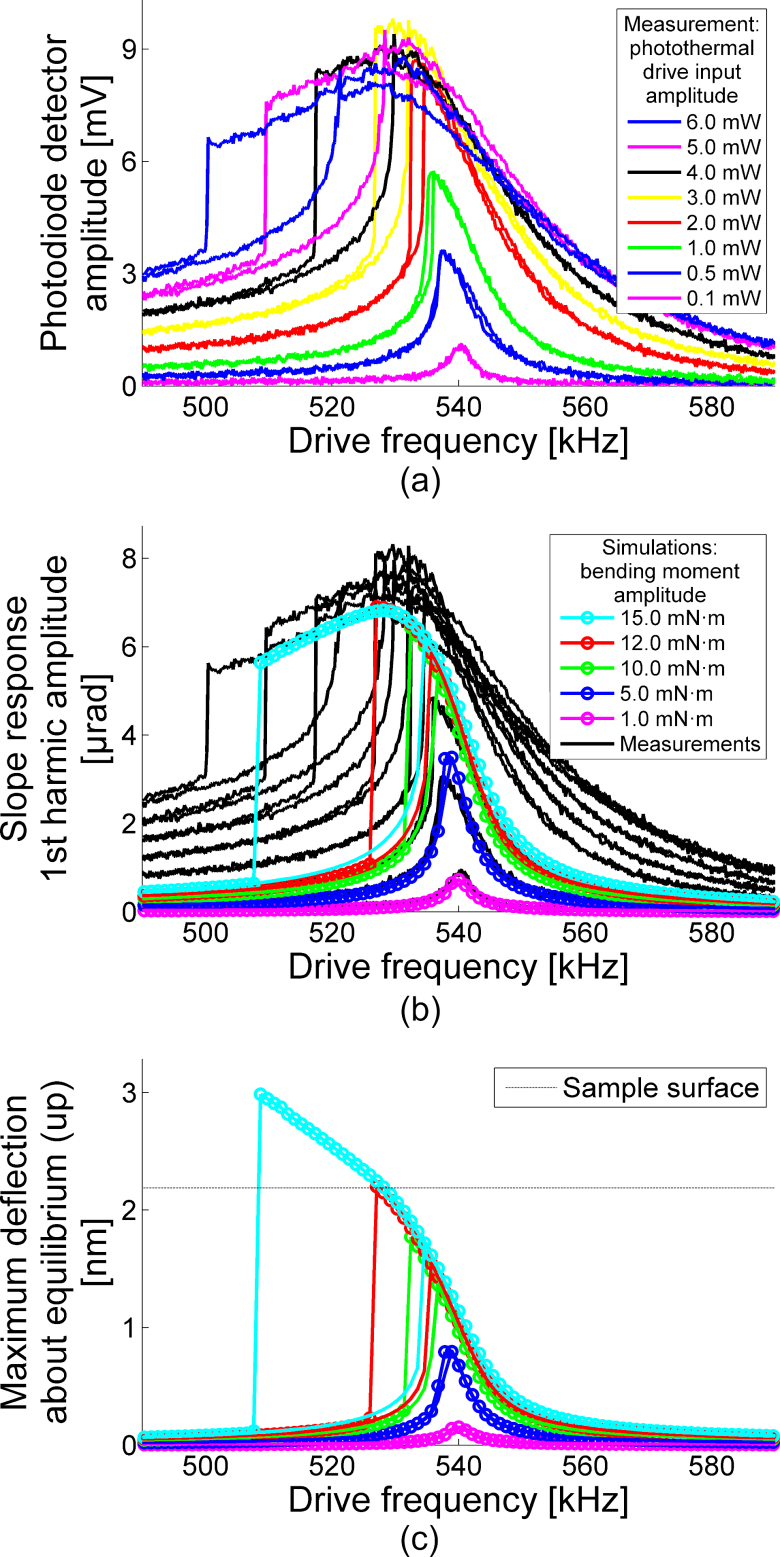
(a) AFM frequency sweep measurements depicted by photodiode detector amplitudes versus photothermal excitation frequency. Colored curves (sweep up and down) identify various photothermal excitation input amplitudes. (b,c) Dynamic simulations of cantilever driven about contact resonance frequency using ten free cantilever eigenfunctions as a basis set. Measurement data is converted to slope response and depicted as black curves. Simulation data points are shown as colored circles, closed for increasing frequency sweep and open for decreasing, with lines connecting to elucidate jumps between periodic solution branches for both slope response first harmonic amplitudes (b) and maximum probe deflection about equilibrium (c). The undeformed sample surface location is indicated as a threshold for tip–sample detachment.

Experimental measurements and numerical simulations of cantilever response amplitude as a function of drive frequency at different drive amplitudes are shown in Figure 1. Figure 1a shows the experimental photodiode amplitude signal as recorded with the lock-in amplifier of the AFM in units of volts. Figure 1b compares the photodiode amplitude signal in units of μrad to a numerical simulation of the cantilever response driven by moment couples of different magnitudes. Figure 1c shows the maximum deflection of the cantilever about static equilibrium for the numerical simulations. The dashed grey line in this Figure indicates the location of the undeformed sample surface. These results directly compare the numerical simulations with experimental measurements.

**Figure 2:**
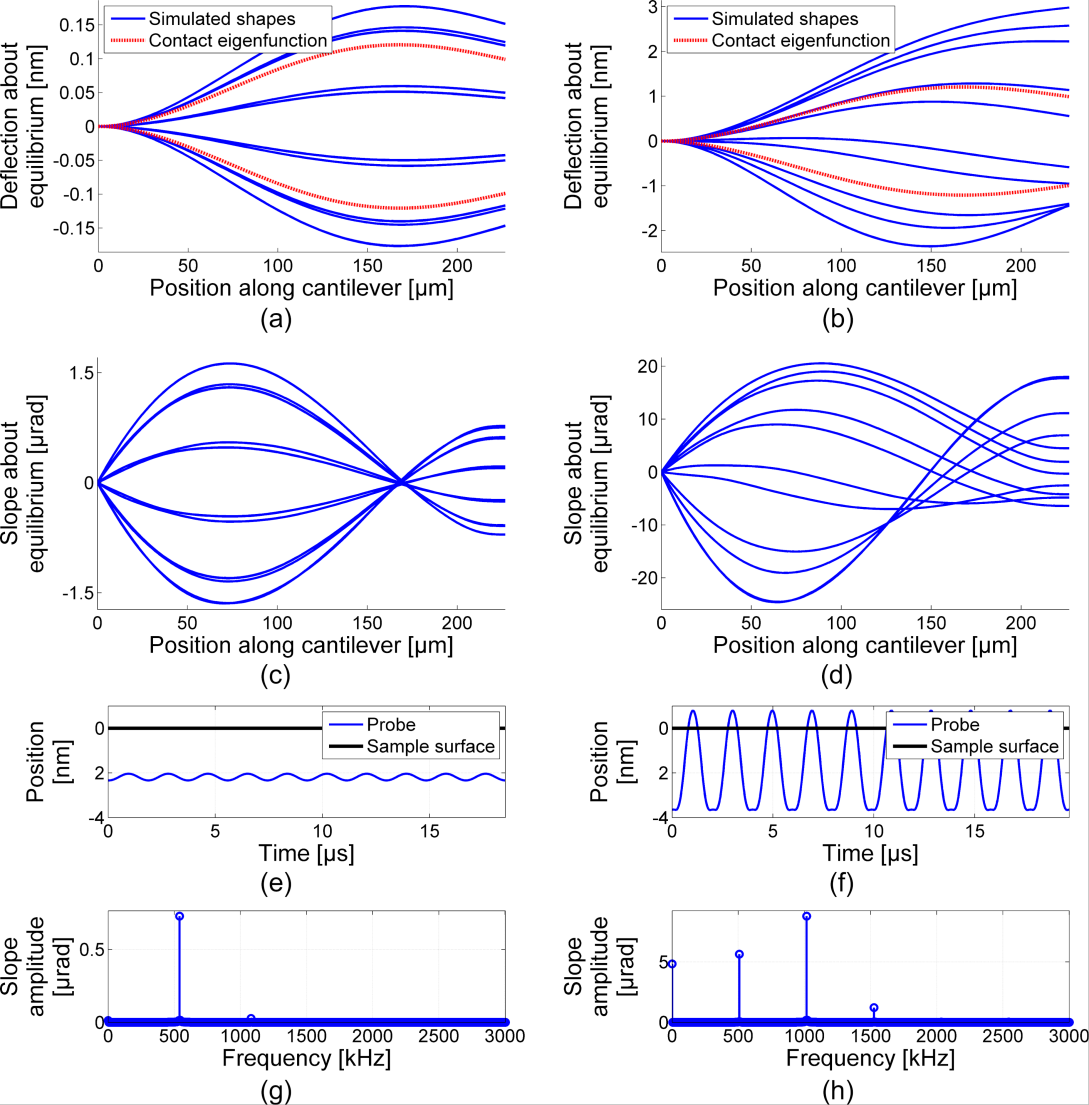
Detailed dynamic behavior of two numerical simulations at steady-state and at resonance: low bending moment amplitude of 1 N·m at 540 kHz (left column), and high bending moment amplitude of 15 N·m at 508.7 kHz (right column). (a, b) Stroboscopic depiction of cantilever deflection and (c, d) slope shapes about static equilibrium (blue, solid), with linear eigenfunction of cantilever–sample system (red, dashed) overlay. (e, f) Probe tip path in time with depiction of the undeformed sample surface location for reference. (g, h) Frequency domain FFT of slope response to connect with direct observables in CR-AFM measurements.

Detailed breakdowns of the numerical simulations for two different moment couples are shown in Figure 2. The lowest bending moment couple amplitude of 1 N·m is shown in the left column (Figure 2a,c,e,g), and the highest bending moment couple amplitude of 15 N·m is shown in the right column (Figure 2b,d,f,h). Figure 2a and Figure 2b represent stroboscopic depictions of cantilever deflection, and Figure 2c and Figure 2d represent stroboscopic depictions of the cantilever slope. For each period of steady-state oscillation, ten equispaced snapshots about the static equilibrium of the cantilever are provided. The deflection plots include additional shapes for the analytical first eigenfunction of the cantilever–sample system. Figure 2e and Figure 2f are plots of the steady-state oscillation position of the probe tip versus time, with the undeflected sample surface location provided for reference. Figure 2g and Figure 2h are the accompanying fast Fourier transform (FFT) data for the steady-state oscillation slope response of the cantilever at the location above the probe tip. Note that the first harmonic amplitude (i.e., the FFT amplitude corresponding to the applied drive frequency) is the value plotted for the simulations in Figure 1b since it corresponds to the response amplitude of the photodiode detector from the lock-in amplifier in the experimental measurements.

**Figure 3:**
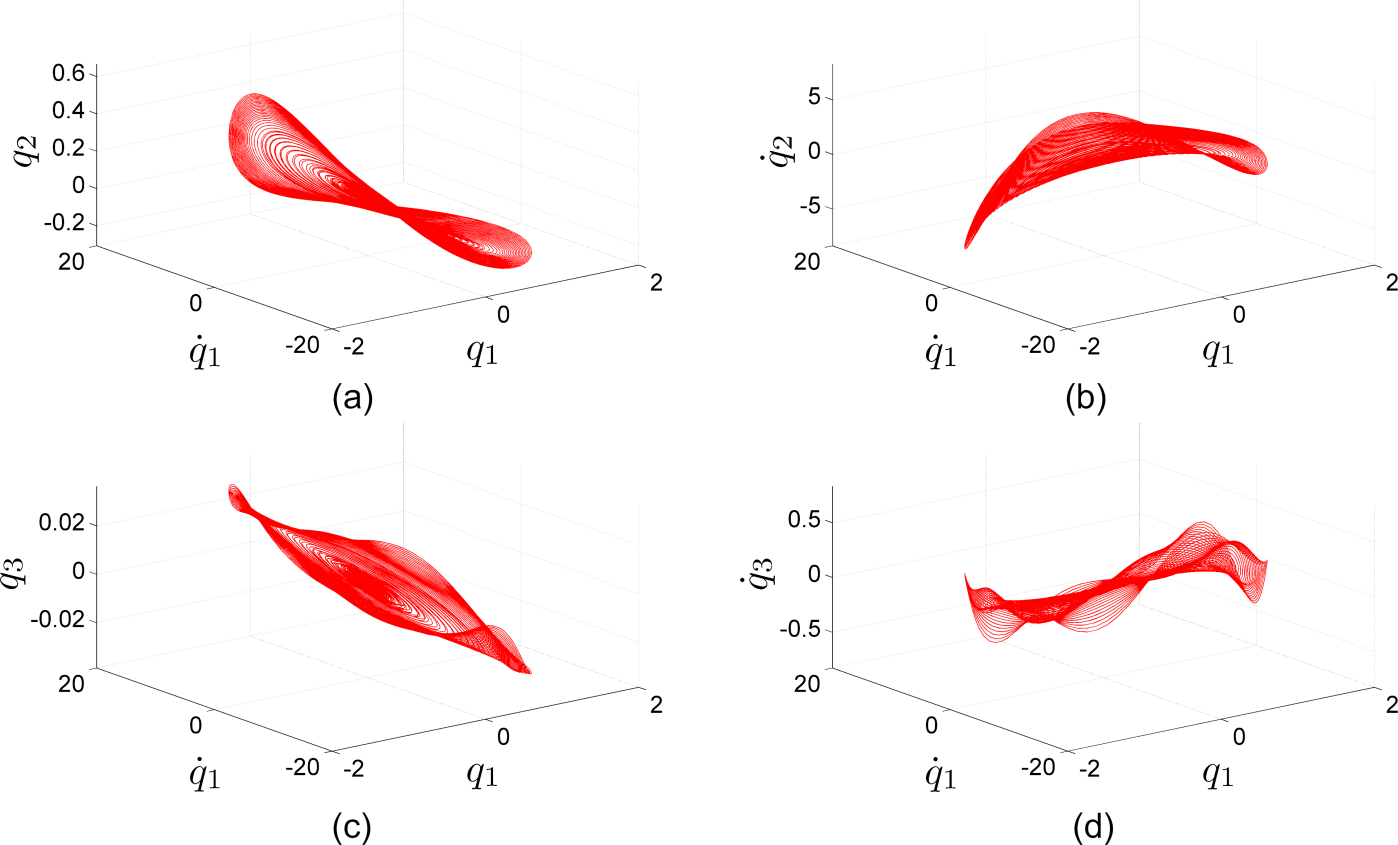
Steady-state periodic orbits (nonlinear normal modes) from simulations in Figure 1 projected onto phase space comprised of state variables. (a) Second basis function displacement *q*_2_ and (b) velocity 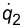, as well as (c) third basis function displacement *q*_3_ and (d) velocity 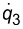 are plotted against first basis function displacement *q*_1_ and velocity 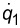.

We also explore the aggregate numerical simulation data of Figure 1 by way of the phase space representation in Figure 3. The phase space has as many dimensions as state variables, but we restrict our viewing to the dominant contributors of the first three basis function modal coordinates 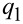, 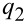, 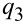 and their respective time derivatives 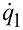, 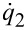, 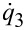. Figure 3a to Figure 3d are all plotted with 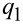 and 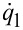 in the horizontal plane along with 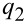, 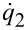, 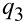, and 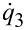 on the vertical axis, respectively. Each closed loop is a steady-state periodic orbit corresponding to an operating amplitude and frequency.

## Discussion

The experimental measurements in Figure 1a reveal interesting features in the dynamic behavior of the cantilever as it is driven by increasing the harmonic excitation amplitude. At the lowest drive amplitude, the response amplitude frequency sweep displays linear behavior, that is, a symmetric resonance peak over the first contact resonance frequency. With increasing drive amplitude, the response amplitude of the resonance peak increases, but the frequency of the resonance peak decreases, that is, a nonlinear softening effect. Once a certain drive amplitude threshold is crossed (approx. 3.0 mW photothermal excitation input amplitude), the response amplitude of the resonance peak itself climaxes and then decreases with increasing drive amplitude. Explaining this final feature in connection to cantilever motion is the main target of our investigation.

Simulations of the cantilever–sample system in Figure 1b and Figure 1c allow us to look behind the AFM measurement features and into the accompanying dynamics at play. The cantilever slope first harmonic amplitude frequency sweeps in Figure 1b match the on-resonance behavior shown in the experimental measurements (black background lines). There is a linear response at low drive amplitude, a nonlinear softening at moderate drive amplitude, and a climax followed by a decrease in response amplitude at high drive amplitude. Comparing this to the features of the maximum displacements in Figure 1c throughout the same simulated frequency sweeps, we notice that the climax of the cantilever slope first harmonic amplitude coincides with the onset of tip–sample detachment, which is consistent with prior works [26–28]. Furthermore, though the linear and nonlinear softening features are represented in the maximum deflection, the climax and decrease in response amplitude are not.

To understand why this difference in features between slope and deflection exist, we first look to the deflection and slope shapes of Figure 2a to Figure 2d. The low operating amplitude deflection shapes (blue solid lines) in Figure 2a appear to be nearly identical to the analytical first eigenfunction of the cantilever–sample system (red dashed lines). When compared to the slope shapes of Figure 2c, one might expect that a scalar quantity could be assigned to map from slope to deflection at any point along the length of the cantilever. This is the standard approach for extracting deflection measurements in most AFM operating modes. However, at high operating amplitudes of Figure 2b and Figure 2d, we see that such a scalar connection is no longer preserved. Interestingly, the slope values near the free end of the cantilever have the same sign at both extremes of the steady state motion, so a mapping from slope to deflection at points in this region is no longer unique.

We find further explanation of the discrepancy between slope and displacement through the frequency space breakdown of cantilever motion at the location of the probe tip shown in Figure 2e to Figure 2h. The low operating amplitude probe tip path in Figure 2e appears to be a single sinusoid, and since we have seen that deflection and slope about static equilibrium share a scalar relationship, this is confirmed in the frequency domain (FFT) of the slope response in Figure 2g. At high operating amplitude near resonance in Figure 2f, the probe tip motion is more complex and even paths above the sample surface. Not surprisingly, the frequency domain of the slope amplitudes echo the complexity and reveal higher harmonics, or dynamics at multiples of the operating frequency, at play. This is crucial because the lock-in amplifier used to monitor the photodiode output during frequency sweeps is set to only observe dynamics coinciding with the drive frequency. In the standard measurement configuration, any other frequency content, for example higher harmonics of cantilever motion, are not observed.

Finally, we turn to nonlinear dynamics for an additional perspective on the behavior. In a recent unification of nonlinear normal mode (NNM) definitions, Haller and Ponsioen state that a NNM is a “recurrent motion with a discrete Fourier spectrum of […] frequencies” for general dissipative systems [29]. This is a relaxation of prior NNM definitions from Rosenberg [30] and Shaw and Pierre [31] that applies to a broader scope of dynamical systems and encompasses fixed points, periodic orbits, and invariant tori realizations of NNMs. In the simulations of Figure 1 projected onto the phase space sets of Figure 3, each steady-state periodic orbit is a NNM corresponding to a specific operating amplitude and frequency. We can consider the collection of simulated NNMs as a family of periodic orbits (FPO), which appears to be constrained to a manifold in phase space that can be parametrized by the state variables 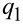 and 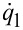 of the first basis function. While NNM analysis in prior literature can be employed to investigate the transient dynamics settling to individual NNMs of the FPO, the FPO itself may be a useful tool in model order reduction for steady-state periodic orbit analysis to reduce a model from many degrees of freedom (e.g., ten in simulations of Figure 1 and Figure 3) to few or one (e.g., the first basis function of state variables 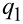 and 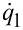).

Tip–sample detachment is detrimental to CR-AFM measurements and imaging. Techniques that attempt to use cantilever amplitude to predict material properties depend on a unique mapping between the photodiode detector amplitude and the cantilever deflection amplitude, which is seen to erode at increasing operating amplitude near resonance. Even when the resonance frequency is the only observable of interest, tracking of it via both phase-locked loops (PLL) and dual A/C resonance tracking (DART) suffer from the presence of detachment by incorrectly identifying the fundamental contact resonance frequency due to the severe nonlinearities present. Last, the AFM probe tip likely deforms at an expedited rate as it is hammering against the surface during tip–sample detachment cycles, which negatively affects property measurements depending on constant tip geometry.

## Conclusion

**Figure 4:**
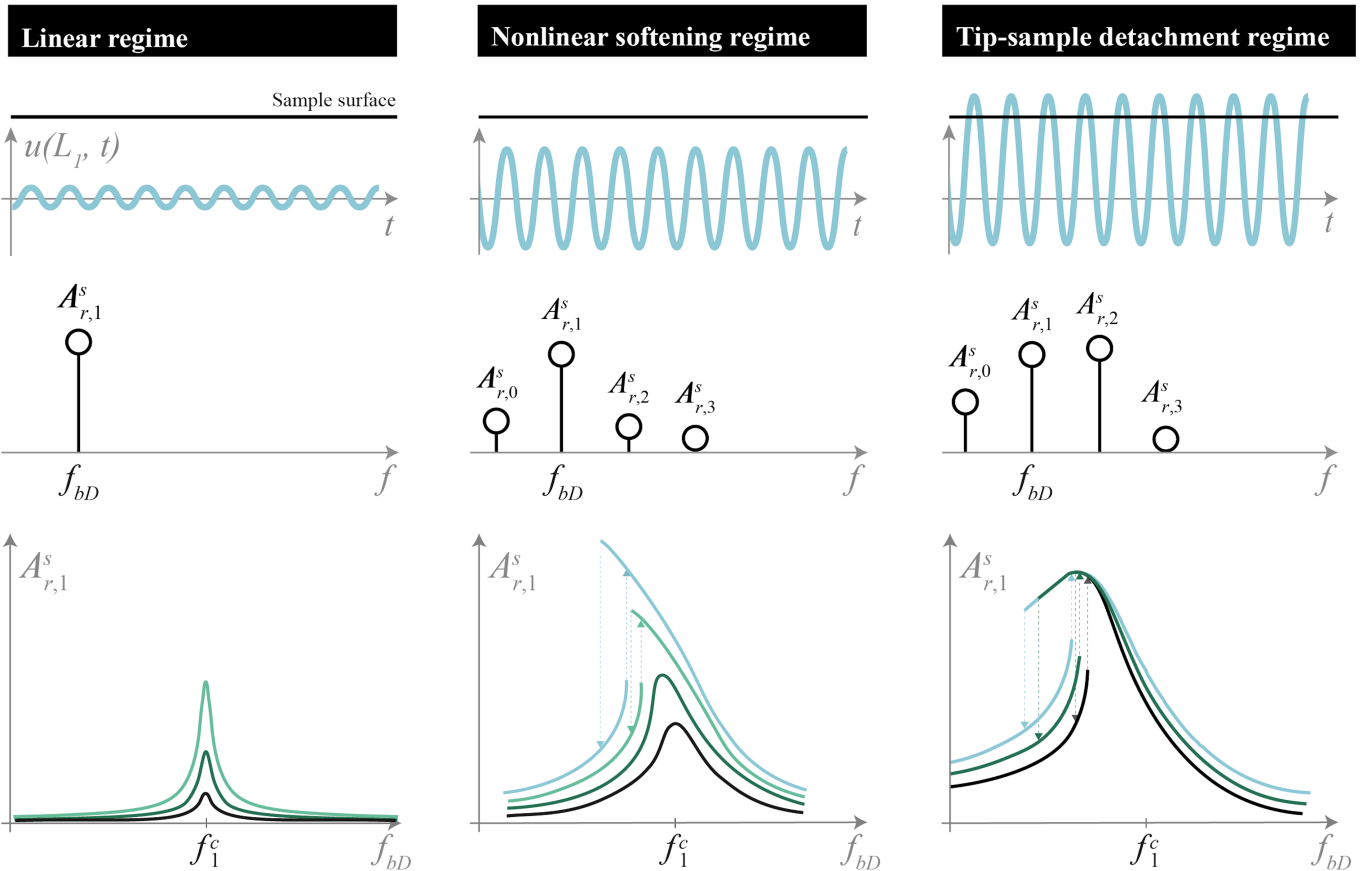
Infographic for summary of linear, nonlinear softening, and tip–sample detachment regimes for qualitative AFM cantilever behavior corresponding to low, moderate, and high drive amplitude excitation, respectively. The top plots depict representations of the AFM probe tip path in time indented below sample surface, the middle plots show representations of harmonic contributions to slope response amplitude of cantilever above probe tip, and the bottom plots provide representations of slope response first harmonic amplitudes versus drive frequencies at various drive amplitudes.

In summary, CR-AFM users must be aware of the potential for detachment of the tip from the sample. Without the ability observe the interaction between the AFM probe tip and the sample directly, users must infer based on the motion of the cantilever. We are able to simulate the cantilever dynamics and obtain quantitative matching with experimental, on-resonance behavior of tip–sample detachment. Figure 4 provides a guide for the different regimes of linear, nonlinear softening, and tip–sample detachment that can be observed while taking measurements or imaging. In the linear regime of low operating amplitude, the probe tip remains indented into the sample; it oscillates as a single-frequency sinusoid, and resonance peaks do not shift in frequency with varying operating amplitudes. At moderate operating amplitude, though the probe tip may remain in contact with the sample, the presence of higher harmonics and nonlinear softening of the resonance peak distorts measurements and should be taken as a signal of caution. If the operating amplitude continues to increase, it may exceed a threshold above which detachment can occur. This tip–sample detachment regime is observable via monitoring of the cantilever motion and identified by the characteristic climax (and, in this case, decrease) of resonance peak in the slope response first harmonic amplitude frequency sweeps. Detachment renders standard CR-AFM, PFM, or ESM analysis techniques invalid for data interpretation and may even result in more rapid probe tip damage as it impacts the sample surface.

## Materials and Methods

### AFM measurements

**Figure 5:**
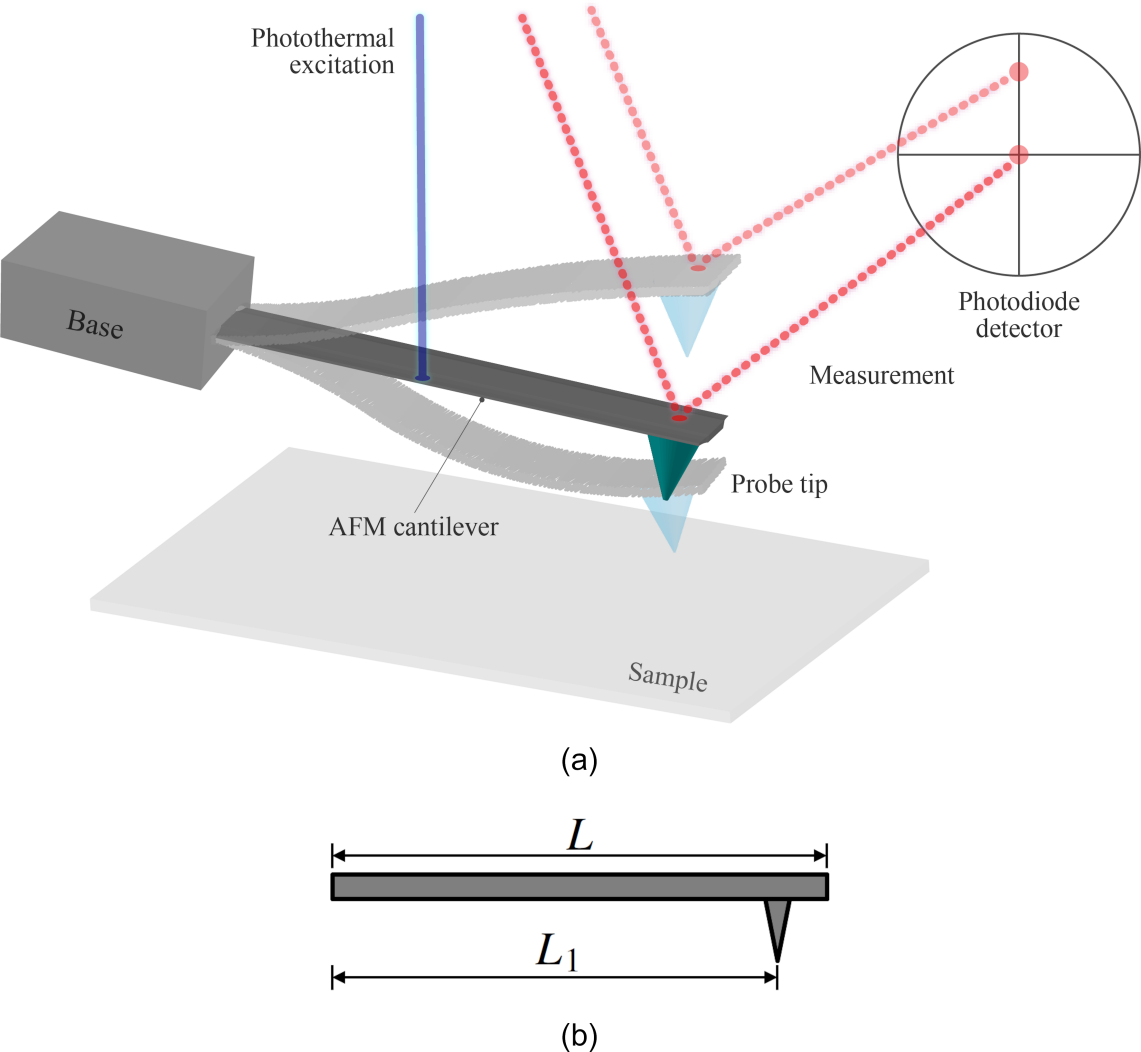
(a) CR-AFM schematic for equipment layout depicting the key components for excitation and measurement. The latter of which is performed with a laser reflecting off of the top surface of the cantilever and onto a photodiode detector. Transparent AFM cantilever shapes are included to suggest the motion of the device during vibration response. Though the laser spot change on the detector appears to the naked eye as a resultant of the cantilever deflection, the vertical scaling is greatly exaggerated for clarity; it is actually the slope change of the cantilever which significantly dominates the detection. (b) AFM cantilever side view with total length *L* and location of the probe tip labeled *L*_1_.

An AFM utilizes a nanometer-scale microscopy technique that consists of a sharp tip mounted on a microcantilever to probe a sample surface, as shown in Figure 5a. Measurements were conducted using a Cypher S AFM microscope (Asylum Research, an Oxford Instruments Company, Santa Barbara, CA, USA) with a NCLAu AFM cantilever (NANOSENSORS, Neuchatel, Switzerland) on a silicon sample. This AFM system is equipped with Asylum Research’s blueDrive photothermal excitation module for laser-based excitation of the cantilever.

The optical lever sensitivity (OLS) corresponding to the static cantilever beam shape with a point load at the tip was calculated from a force displacement curve [32] on the silicon sample. The thermal method [33] was then used to calculate the static cantilever bending stiffness. To study the process of tip detachment, we swept drive frequencies (low to high, then high to low) at selected drive amplitudes near the first contact resonance frequency of the cantilever–sample system. The cantilever position was recorded with an optical lever system consisting of a detection laser and a quadrant photodiode. The photodiode signal was converted into amplitude and phase at the drive frequency using a lock-in amplifier.

The OLS resulting from the above calibration procedure is only valid for quasistatic bending of the cantilever or the very similar shape of the first freely vibrating eigenmode. It does not apply to the surface-coupled resonances that are the focus of this study. Optical lever systems are predominantly sensitive to changes in cantilever slope. Since the cantilever is deforming in a known shape during the OLS calibration, it is only a matter of multiplying the observed OLS by a ratio of the deflection to slope for a cantilever deforming under a point load applied at the location of the probe tip (assumed from manufacturer specifications) to extract a calibration parameter that can convert between photodiode voltage and cantilever slope. This provides a calibration parameter that remains valid in all measurement circumstances.

### Analytical model

The core of the computational exploration is the partial differential equation (PDE) governing the dynamics of the AFM cantilever. At rest, the equilibrium indentation Δ* is defined as:


[1] $$\Delta *=-w*\left(L_{1}\right)-Z,$$

where w*(*L*_1_) is the equilibrium deflection of the cantilever at the location of the probe tip *L*_1_, shown in Figure 5b, and *Z* is the undeflected tip position (upward) relative to the undeformed sample surface. While vibrating, the dynamic indentation Δ_dyn_ is defined as:


[2] $$\Delta_{\text{dyn}}=\Delta *-u\left(L_{1},t\right),$$

where *u*(*L*_1_,*t*) is the deflection of the cantilever about the equilibrium at the location of the probe tip. The tip–sample interaction *F*_TS_ is rooted in the Derjaguin, Muller, and Toporov (DMT) model of adhesive contact between particles [34], but modified via works by Shaik et al. [35] to linearize the adhesive regime and Labuda et al. [36] to replace the standard Hertzian indentation model by a generalized Sneddon law interaction for a variety of tip geometries [37]. It is defined as:


[3]

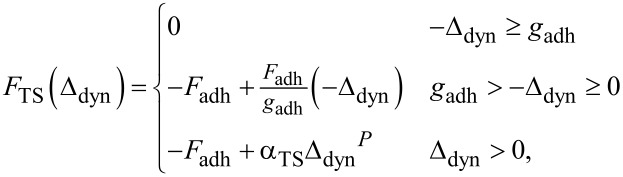



where *F*_adh_ is the maximum adhesive force, *g*_adh_ is the range of the linear attractive region, α_TS_ is the indentation coefficient, and *P* is the parameter controlling the probe tip geometry. Photothermal excitation of the AFM cantilever is approximated as a pair of opposing bending moments centered at the laser spot location *L*_bD_, measured from the base of the cantilever, and separated from each other by the laser spot diameter (double the laser spot radius *r*_bD_). Each of the two bending moments is harmonic with amplitude *M*_bD_ and frequency Ω_bD_. Altogether, the equation of motion PDE is assembled as:


[4]

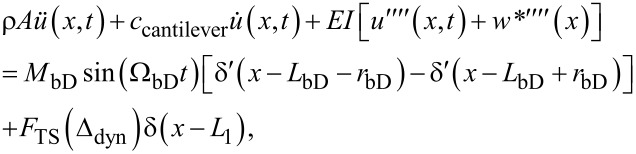



where *u*(*x*,*t*) is the cantilever deflection at position *x* and time *t* about equilibrium, w*(*x*) is the equilibrium shape of the cantilever, ρ*A* is the mass per unit length, *c*_cantilever_ is the linear viscous damping coefficient, and *EI* is the cantilever flexural rigidity. Dots over variables refer to time derivatives, while primes denote partial derivatives with respect to the position along the cantilever length.

### Parameter identification

Careful identification of system parameters is critical for a meaningful comparison of simulations to measurements. Fortunately, much of what is needed is common practice to AFM users, and the following sequence steps through extraction of relevant of quantities predominantly via techniques described by Hurley and co-workers [1]. These are collected for reference in Table 1. First, linear vibration cantilever modes are chosen as an orthogonal basis set upon which to project the PDE, defined as [38]:


[5]

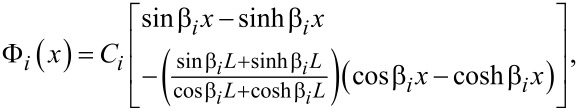



and normalized by Φ*_i_*(*L*_1_) = 1 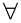 *i* = 1,2,3,…,*N*, where *N* is the number of basis functions used in the discretization set, and *C_i_* is the basis coefficient used to satisfy such normalization. The basis set is used to separate the deflection variable into spatial and temporal portions as follows:


[6] $$u\left(x,t\right)\approx \sum_{i}^{N}\Phi_{i}\left(x\right)q_{i}\left(t\right),$$

where *q_i_*(*t*) is the time-varying modal coefficient of the *i*th basis function. This discretization step is pertinent not only to parameter identification, but also the dynamic simulations.

**Table 1:** Parameter identification from AFM measurements.

Parameter	Measured	Parameter	Assumed	Parameter	Calculated

*k*_1,free_	32.8 N/m	*L*_1_	225 μm	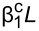	3.413
*f*_1_	163 kHz	β_1_*L*	1.875	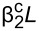	5.180
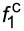	540 kHz	*L*_bD_	0.1 · *L*	*L* (γ = *L*_1_/*L*)	227 μm (0.99)
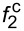	1244 kHz	*r*_bD_	1 μm	*k*_ratio_	16.4
setpoint	0.2 V			*k*_cantilever_	31.9 N/m
InvOLS	1.27 × 10−7 m/V			*EI*	1.2 × 10−10 N·m2
*Q*	174	Parameter	Tuned	*k*sample	524.0 N/m
				
*g*Adh	14 nm	*P*	1.4	ρ*A*	5.4 × 10−7 kg/m
*F*Adh	9.2 nN			*c*cantilever	0.018 N·s/m
*F*TS*	810 nN			αTS	1.1 × 106 N/m*P*
				Δ*	2.19 nm

Measurements of the resonance frequencies for both the free and contact cases allow us to identify critical stiffness quantities required for converting raw experimental output into physical quantities. The dispersion relation for wave number and frequency is given as:


[7] $${\left(\beta_{n}L\right)}^{2}=2\pi f_{n}\sqrt{\frac{\rho AL^{4}}{EI}},$$

where β*_n_* and *f_n_* are the wavenumber and resonance frequency of the *n*th mode, respectively [38]. Note that this is also valid for the cantilever in contact with the sample using the contact wavenumber 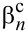 and contact resonance frequency 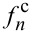 of the *n*th mode. As such, a ratio can be constructed between the first free vibration mode and the first contact vibration mode:


[8] $$\left(\beta_{n}^{\text{c}}L\right)=\left(\beta_{1}L\right)\sqrt{\frac{f_{n}^{\text{c}}}{f_{1}}}.$$

This is used to back out 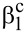
*L* and 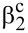 from the measured frequencies *f*_1_, 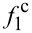, and 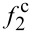 as well as the known β_1_*L* = 1.875 from free vibration theory of cantilevers [38]. By defining the relative tip position ratio as:


[9] $$\gamma =\frac{L_{1}}{L},$$

and the relative spring constant ratio as:


[10] $$k_{\text{ratio}}=\frac{k_{\text{sample}}}{k_{\text{cantilever}}},$$

where *k*_sample_ and *k*_cantilever_ are the equivalent linear spring constants of the sample at equilibrium and the cantilever, respectively, we assemble the characteristic equation as [39]:


[11] $$k_{\text{ratio}}=\frac{2}{3}{\left(\beta_{n}^{\text{c}}L\gamma \right)}^{3}\frac{1+\cos\beta_{n}^{\text{c}}L\cosh\beta_{n}^{\text{c}}L}{D_{\text{r}}},$$

where


[12] $$\begin{array}{c} D_{\text{r}}=\left[\begin{array}{l} \sin\beta_{n}^{\text{c}}L\left(1-\gamma \right)\cosh\beta_{n}^{\text{c}}L\left(1-\gamma \right)\\ -\cos\beta_{n}^{\text{c}}L\left(1-\gamma \right)\sinh\beta_{n}^{\text{c}}L\left(1-\gamma \right) \end{array}\right]*\\ \left(1-\cos\beta_{n}^{\text{c}}L\gamma \cosh\beta_{n}^{\text{c}}L\gamma \right)\\ -\left(\sin\beta_{n}^{\text{c}}L\gamma \cosh\beta_{n}^{\text{c}}L\gamma -\cos\beta_{n}^{\text{c}}L\gamma \sinh\beta_{n}^{\text{c}}L\gamma \right)*\\ \left[1+\cos\beta_{n}^{\text{c}}L\left(1-\gamma \right)\cosh\beta_{n}^{\text{c}}L\left(1-\gamma \right)\right]. \end{array}$$

This relationship has a unique physically relevant solution valid for both the first and second contact modes, thus determining *k*_ratio_ and γ for the system. We define the cantilever spring constant as [1]:


[13] $$k_{\text{cantilever}}=\frac{3EI}{L_{1}{}^{3}},$$

and the first free vibrating mode stiffness as [40]:


[14] $$k_{\text{1,free}}=EI\int_{0}^{L}\Phi_{1}\left(x\right){{{{\Phi^{\prime }}^{\prime }}^{\prime }}^{\prime }}_{1}\;\left(x\right)\text{d}x,$$

allowing us to solve for *k*_cantilever_ and the flexural rigidity *EI*. Combining these with previous parameter results allow for the calculation of the sample stiffness *k*_sample_ and the mass per unit length ρ*A*. Next, combining the latter term with the quality factor *Q* of the first contact mode, the linear viscous cantilever damping is defined as:


[15] $$c_{\text{cantilever}}=\frac{2\pi f_{1}^{\text{c}}}{\rho AQ}.$$

Remaining system parameters relating to the tip–sample indentation model of Equation 3 are defined in conjunction with experimental observables. The adhesion force *F*_adh_* and adhesion gap *g*_adh_ are identified from the retraction force–distance curve taken just before vibration sweeps. While *g*_adh_ is observed directly, *F*_adh_ requires multiplication with *k*_cantilever_ and the inverse OLS to convert from voltage to force. Similarly, the equilibrium tip–sample force *F*_TS_* uses the same conversion to interpret the setpoint voltage as a force. Finally, the indentation power *P* is tuned to match the softened resonance frequency at the response amplitude climax of Figure 1a, allowing for the subsequent calculation of the coefficient α_TS_, equilibrium indentation Δ*, and undeflected tip position *Z*.

#### Simulations

All simulations were conducted in MATLAB using an ordinary differential equation solver to evaluate time-marching integration. For each frequency sweep set in Figure 1, the system was simulated at a selected bending moment couple drive amplitude until steady-state, at which point the frequency was stepped incrementally up or down using final state variable values from the previous simulation point as initial conditions. A fast Fourier transform of the steady-state time-domain slope response of the AFM cantilever at the location of the probe tip is used to extract the first harmonic amplitude for comparison with measurements. Using the full cantilever response, stroboscopic sampling throughout a period provides explanation of the shapes experienced during simulations. Lastly, orbits of the state variables *q_i_* and 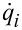 for *i* = 1,2,3 are plotted to examine evidence and behavior of NNMs.

## Supporting Information

Additional AFM measurements identifying the tip–sample detachment signature are provided in Supporting Information File 1. These were performed using the same Cypher S AFM microscope with an ACLA AFM cantilever (Applied NanoStructures, Inc., Mountain View, CA, USA) on a silicon sample, but driven by a piezoelectric actuator under the sample instead of via photothermal excitation. This leads to less ideal (“forest of peaks”) transfer functions and can complicate parameter identification [41–42].

A second simulation-only example with hypothetical parameter values is provided in Supporting Information File 2. The associated NNMs are collected into a FPO like those of Figure 3.

File 1 Additional AFM measurements.

File 2 Hypothetical simulations with NNMs.
