# Supplementary material for: Cantilever signature of tip detachment during contact resonance AFM
Source: Beilstein J Nanotechnol. 2021 Nov 24;12:1286–96. doi: 10.3762/bjnano.12.96 (PMC8630435; doi:10.3762/bjnano.12.96)
Supplement: File 1 — Additional AFM measurements. [file Beilstein_J_Nanotechnol-12-1286-s001.pdf]

BEILSTEIN JOURNAL OF NANOTECHNOLOGY

Supporting Information

for

Cantilever signature of tip detachment during contact resonance AFM

Devin Kalafut, Ryan Wagner, Maria Jose Cadena, Anil Bajaj and Arvind Raman

Beilstein J. Nanotechnol. **2021,** *12,* 1286–1296. doi:10.3762/bjnano.12.96

Additional AFM measurements

License and Terms: This is a supporting information file under the terms of the Creative Commons Attribution License (https://creativecommons.org/licenses/by/4.0). Please note that the reuse, redistribution and reproduction in particular requires that the author(s) and source are credited and that individual graphics may be subject to special legal provisions.
The license is subject to the *Beilstein Journal of Nanotechnology* terms and conditions: (https://www.beilstein-journals.org/bjnano/terms)

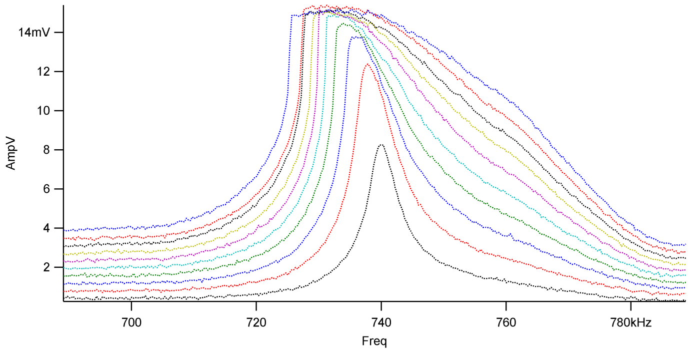

(a)

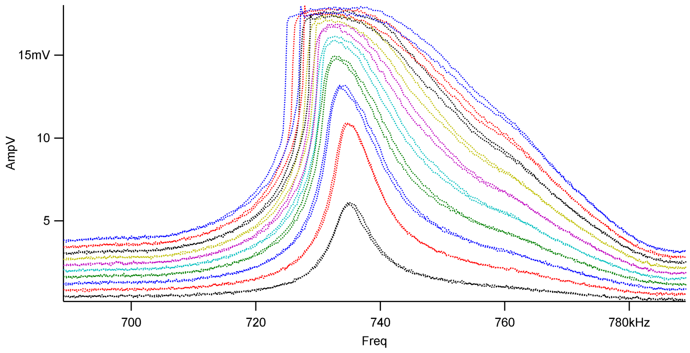

(b)

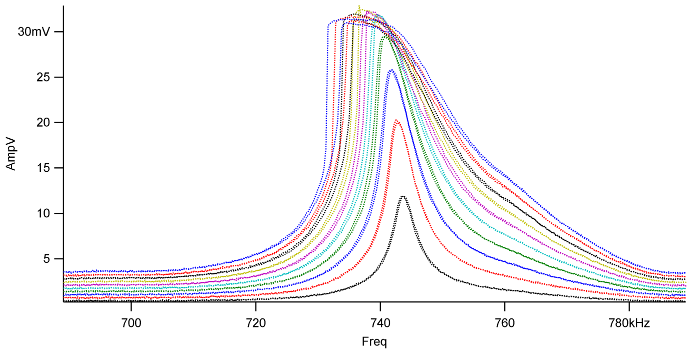

(c)

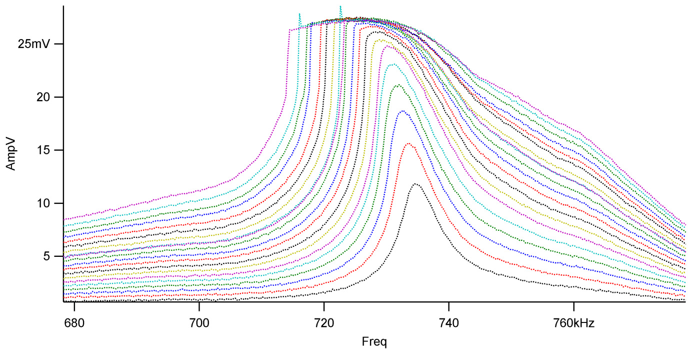

(d)

Figure S1: Additional evidence of tip-sample detachment on silicon samples at various piezoelectric drive amplitudes, shown as photodiode detector amplitude vs drive frequency.
