# Supplementary material for: Cantilever signature of tip detachment during contact resonance AFM
Source: Beilstein J Nanotechnol. 2021 Nov 24;12:1286–96. doi: 10.3762/bjnano.12.96 (PMC8630435; doi:10.3762/bjnano.12.96)
Supplement: File 2 — Hypothetical simulations with NNMs. [file Beilstein_J_Nanotechnol-12-1286-s002.pdf]

BEILSTEIN JOURNAL OF NANOTECHNOLOGY

Supporting Information

for

Cantilever signature of tip detachment during contact resonance AFM

Devin Kalafut, Ryan Wagner, Maria Jose Cadena, Anil Bajaj and Arvind Raman

Beilstein J. Nanotechnol. **2021,** *12,* 1286–1296. doi:10.3762/bjnano.12.96

Hypothetical simulations with NNMs

License and Terms: This is a supporting information file under the terms of the Creative Commons Attribution License (https://creativecommons.org/licenses/by/4.0). Please note that the reuse, redistribution and reproduction in particular requires that the author(s) and source are credited and that individual graphics may be subject to special legal provisions.
The license is subject to the *Beilstein Journal of Nanotechnology* terms and conditions: (https://www.beilstein-journals.org/bjnano/terms)

Table S1: Parameter values for hypothetical simulation example

Parameter	Value
L ($\gamma = L_1/L$)	300 μm (0.97)
L_{bD}	$0.1 \cdot L$
h	4 μm
b	20 μm
ρ	2330 kg·m^{-3}
E	169 GPa
ν	0.278
R	10 nm
E_{sample}	10 GPa
ν_{sample}	0.3
F_{Adh}	20 nm
g_{adh}	10 nm
P	1.5 (Hertz)
Q	100
F_{TS}	200 nN

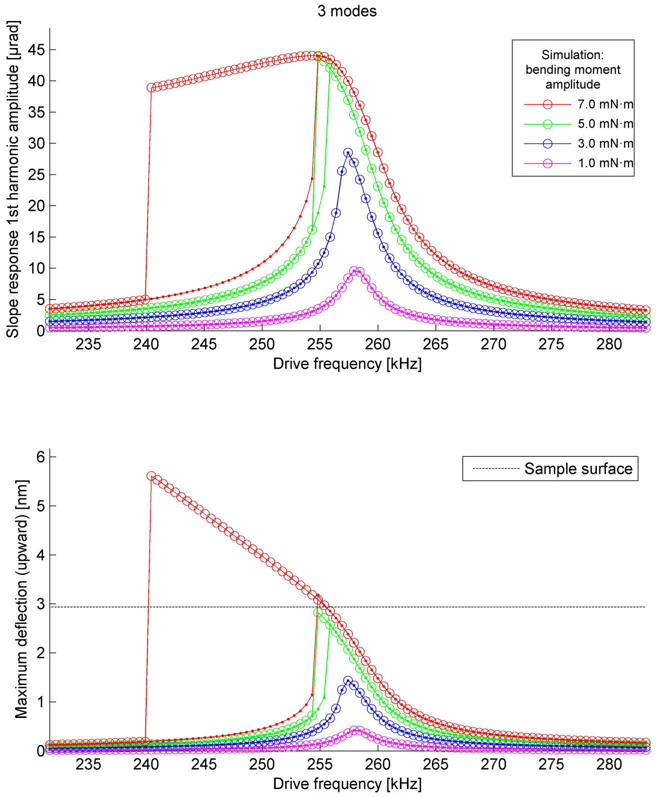

Figure S1: Dynamic simulations of a hypothetical cantilever system (parameter values in Table 1) driven about contact resonance frequency using only three free cantilever eigenfunctions as a basis set. Slope response first harmonic amplitude versus drive frequency (upper) and maximum probe displacement versus drive frequency (lower).

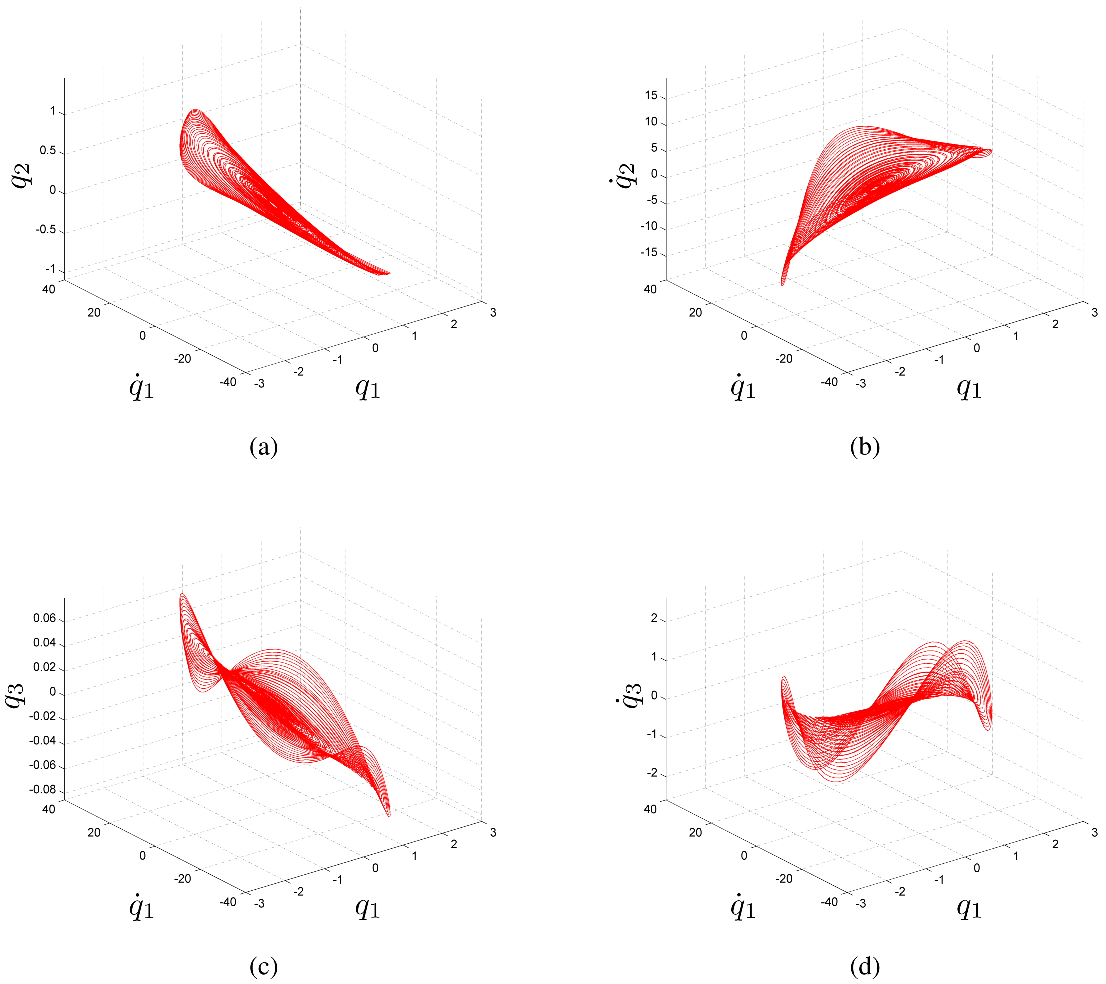

Figure S2: Steady-state periodic orbits (nonlinear normal modes) from hypothetical simulations (parameter values in Table 1) in Figure 1 projected onto phase space comprised of state variables. Second basis function displacement q_2 (a) and velocity $\dot{q}_2$ (b), as well as third basis function displacement q_3 (c) and velocity $\dot{q}_3$ (d) are plotted against first basis function displacement q_1 and velocity $\dot{q}_1$.
